# RSV Vaccine-Enhanced Disease Is Orchestrated by the Combined Actions of Distinct CD4 T Cell Subsets

**DOI:** 10.1371/journal.ppat.1004757

**Published:** 2015-03-13

**Authors:** Cory J. Knudson, Stacey M. Hartwig, David K. Meyerholz, Steven M. Varga

**Affiliations:** 1 Interdisciplinary Graduate Program in Immunology, University of Iowa, Iowa City, Iowa, United States of America; 2 Department of Microbiology, University of Iowa, Iowa City, Iowa, United States of America; 3 Department of Pathology, University of Iowa, Iowa City, Iowa, United States of America; St. Jude Children's Research Hospital, UNITED STATES

## Abstract

There is no currently licensed vaccine for respiratory syncytial virus (RSV) despite being the leading cause of lower respiratory tract infections in children. Children previously immunized with a formalin-inactivated RSV (FI-RSV) vaccine exhibited enhanced respiratory disease following natural RSV infection. Subsequent studies in animal models have implicated roles for CD4 T cells, eosinophils and non-neutralizing antibodies in mediating enhanced respiratory disease. However, the underlying immunological mechanisms responsible for the enhanced respiratory disease and other disease manifestations associated with FI-RSV vaccine-enhanced disease remain unclear. We demonstrate for the first time that while CD4 T cells mediate all aspects of vaccine-enhanced disease, distinct CD4 T cell subsets orchestrate discrete and specific disease parameters. A Th2-biased immune response, but not eosinophils specifically, was required for airway hyperreactivity and mucus hypersecretion. In contrast, the Th1-associated cytokine TNF-α was necessary to mediate airway obstruction and weight loss. Our data demonstrate that individual disease manifestations associated with FI-RSV vaccine-enhanced disease are mediated by distinct subsets of CD4 T cells.

## Author Summary

RSV is a significant healthcare burden and is the leading cause of bronchiolitis and pneumonia during childhood. The failure of the 1960's FI-RSV vaccine trial to not only elicit protection against RSV infection, but also provoke enhanced morbidity and mortality in vaccinees has significantly hampered development of new RSV vaccines for fear of disease potentiation. Therefore we sought to determine the specific immunological mechanisms that mediate FI-RSV VED to provide a framework to evaluate factors associated with disease exacerbation. Work presented herein demonstrate for the first time that individual disease manifestations associated with FI-RSV-immunization are mediated by distinct CD4 T cell subsets and not by eosinophils. Our results stress the need to evaluate multiple disease parameters for future RSV vaccine candidates. Failure to thoroughly assess the immune response and disease manifestations associated with new candidate vaccines may lead to undesired results in vaccine trials and further hinder future vaccine development.

## Introduction

Respiratory syncytial virus (RSV) is the leading cause of hospitalization in infants and young children [1–3]. There is currently no licensed RSV vaccine available. An initial trial in the late 1960's with a formalin-inactivated RSV (FI-RSV) vaccine ended in failure. FI-RSV vaccination not only failed to induce sterilizing immunity against RSV infection, but also resulted in an increased rate of hospitalization and disease severity after a natural RSV infection in the majority of the volunteers including two cases of fatal disease [4–8]. A study examining the two children that died revealed a significant increase in the number of eosinophils present in the lung parenchyma [4]. Mirroring the results of the FI-RSV vaccine trial, FI-RSV immunization also induces a Th2-biased immune response resulting in pulmonary eosinophilia following RSV challenge in multiple animal models [9–12]. Since the presence of an elevated number of eosinophils in both the lung and peripheral blood was highlighted in the initial vaccine trial reports, the development of pulmonary eosinophilia has become a hallmark of the enhanced respiratory disease (ERD) associated with FI-RSV vaccine-enhanced disease (VED) [4–7]. However, re-examination of the human autopsy specimens from the initial FI-RSV vaccine trials revealed only 1–2% of the total cellular infiltrate in the airways were eosinophils [12]. This observation, in conjunction with similar findings in lung sections from FI-RSV-immunized cotton rats, an alternative model of FI-RSV ERD, has raised questions concerning the role eosinophils play during FI-RSV VED [12]. Therefore, it remains unclear if eosinophils directly contribute to the severe immunopathology associated with FI-RSV ERD.

Multiple disease manifestations are associated with FI-RSV VED including weight loss, pulmonary inflammation, mucus hypersecretion and airway obstruction. In addition to eosinophils, previous studies have also implicated a pathogenic role for antibodies induced following FI-RSV immunization in mediating VED following a RSV challenge [13,14]. FI-RSV-immunized mice deficient in the complement component C3 exhibit a significant amelioration of pulmonary histopathology after RSV challenge, implicating a role for immune complexes in VED [13]. In addition, non-neutralizing antibody responses correlate with increases in lung histopathology and airway hyperreactivity associated with FI-RSV VED [14]. Supplementation of TLR agonists during FI-RSV-immunization improves affinity maturation of B cell responses and prevents ERD following RSV challenge [14]. However, it remains unclear which immunological factors directly contribute to critical disease parameters associated with FI-RSV VED. The lack of a detailed mechanistic understanding of the causes of FI-RSV VED has made it difficult to appropriately assess the safety of new RSV vaccine candidates. In order to address this critical knowledge gap, we sought to determine the specific immunological factors responsible for mediating the individual disease parameters most associated with FI-RSV VED. In contrast to the prevailing notion, we demonstrate that eosinophils are not required to mediate any of the characteristic disease manifestations associated with FI-RSV VED. In vivo depletion of CD4 T cells prior to RSV challenge led to significant reductions in all disease parameters assessed. Our results show that a Th2-biased immune response is necessary to mediate airway hyperreactivity and mucus hypersecretion disease parameters. In contrast, the Th1-associated cytokine TNF-α was found to be critical for the induction of airway obstruction and weight loss associated with FI-RSV VED. Our studies demonstrate for the first time that distinct subsets of CD4 T cells orchestrate individual disease parameters associated with FI-RSV VED.

## Results

### FI-RSV-immunized mice develop VED following RSV challenge

**Fig 1:**
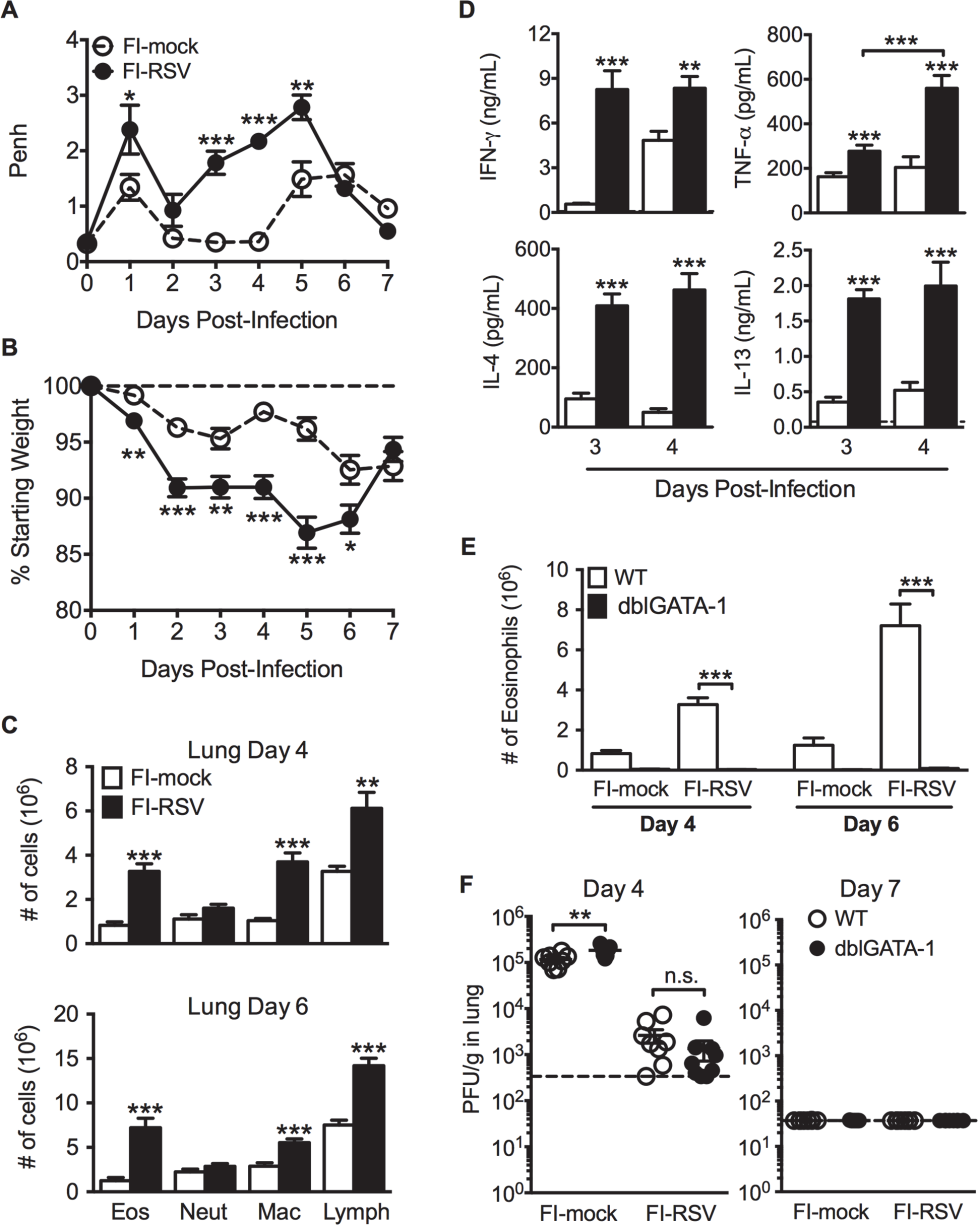
Development of VED following RSV challenge of FI-RSV-immunized mice. (A) FI-mock- and FI-RSV-vaccinated mice were monitored daily for airway obstruction using a whole body plethysmograph. (B) Weight loss was assessed for immunized mice for 7 days following RSV challenge. (C) Total number of eosinophils (CD11c^int^Siglec F^+^), neutrophils (Ly6c^+^Ly6g^hi^), macrophages (CD11c^+^F4/80^+^), and T cell lymphocytes (CD90.2^+^) were quantified from the lungs of vaccinated mice via flow cytometry on day 4 and 6 post-RSV challenge. (D) Cytokine protein amounts in whole lung homogenates from immunized mice were determined on day 3 following RSV infection. Dotted lines indicate the limit of detection. (E) Total number of eosinophils from the lung parenchyma of FI-mock- and FI-RSV-immunized WT and dblGATA-1 mice were quantified on days 4 and 6 following RSV challenge. (F) Plaque assay on lungs from immunized WT and dblGATA-1 mice was performed on days 4 and 7 following RSV challenge. Data are represented as mean ± SEM from two independent experiments (*n* = 12 mice total for A, B, *n* = 8 for C-F). Groups were compared using Student's t-test for two groups or using one-way ANOVA for comparison of more than two groups, * p<0.05, ** p<0.01, *** p<0.001.

ERD was an important clinical manifestation of FI-RSV VED [5–7]. Whole body plethysmography has been previously utilized for the assessment of baseline respiratory patterns that correlate with pulmonary function following viral infections [15,16]. Therefore, we used unrestrained whole body plethysmography to evaluate pulmonary function daily following RSV challenge [17,18] of FI-RSV-immunized BALB/c mice. FI-RSV-primed mice exhibited increased airway obstruction, measured as enhanced pause (Penh, Fig. 1A), during the first five days following RSV challenge when compared to mock-primed controls. Mice vaccinated with FI-RSV also exhibited significantly (p<0.05) increased weight loss (Fig. 1B) compared to the mock-immunized control group between days 1–6 following RSV challenge. The numbers of eosinophils, macrophages and lymphocytes were significantly (p<0.01) increased in the lungs of FI-RSV-immunized mice on days 4 and 6 following RSV challenge (Fig. 1C). Consistent with the induction of pulmonary eosinophilia, FI-RSV-immunized animals exhibit significantly (p<0.001) increased protein amounts of the Th2-associated cytokines IL-4 and IL-13 as compared to mock-immunized controls at days 3 and 4 following RSV infection (Fig. 1D). In addition, there was a significant (p<0.001) increase in both lung IFN-γ and TNF-α protein amounts indicating that the CD4 T cell response consisted of a mixture of Th1 and Th2 cells. A significant difference in IL-17A production was not detected by either ELISA of lung homogenates or following CD4 T cell restimulation and therefore only the Th1- and Th2-associated immune responses were evaluated for the remainder of the study (S1 Fig.).

### Eosinophils are not required to mediate disease parameters associated with FI-RSV VED

The development of pulmonary eosinophilia following RSV challenge of FI-RSV-immunized hosts has become a defining characteristic of RSV VED [4–7]. The increased airway obstruction and weight loss coincide with time points when there is a significant increase in the number of eosinophils in the lung (Fig. 1A-C). To determine the contributions of eosinophils to respiratory disease and weight loss associated with FI-RSV VED, we utilized eosinophil-deficient dblGATA-1 mice [19]. Quantification of eosinophil numbers revealed virtually undetectable numbers of eosinophils in the lung parenchyma (Fig. 1E) of FI-RSV-primed dblGATA-1 mice on day 4 and 6 following RSV challenge. Assessment of viral titers on days 4 and 6 following RSV challenge also showed no difference in lung RSV titers between FI-RSV-immunized WT and eosinophil-deficient mice (Fig. 1F).

**Fig 2:**
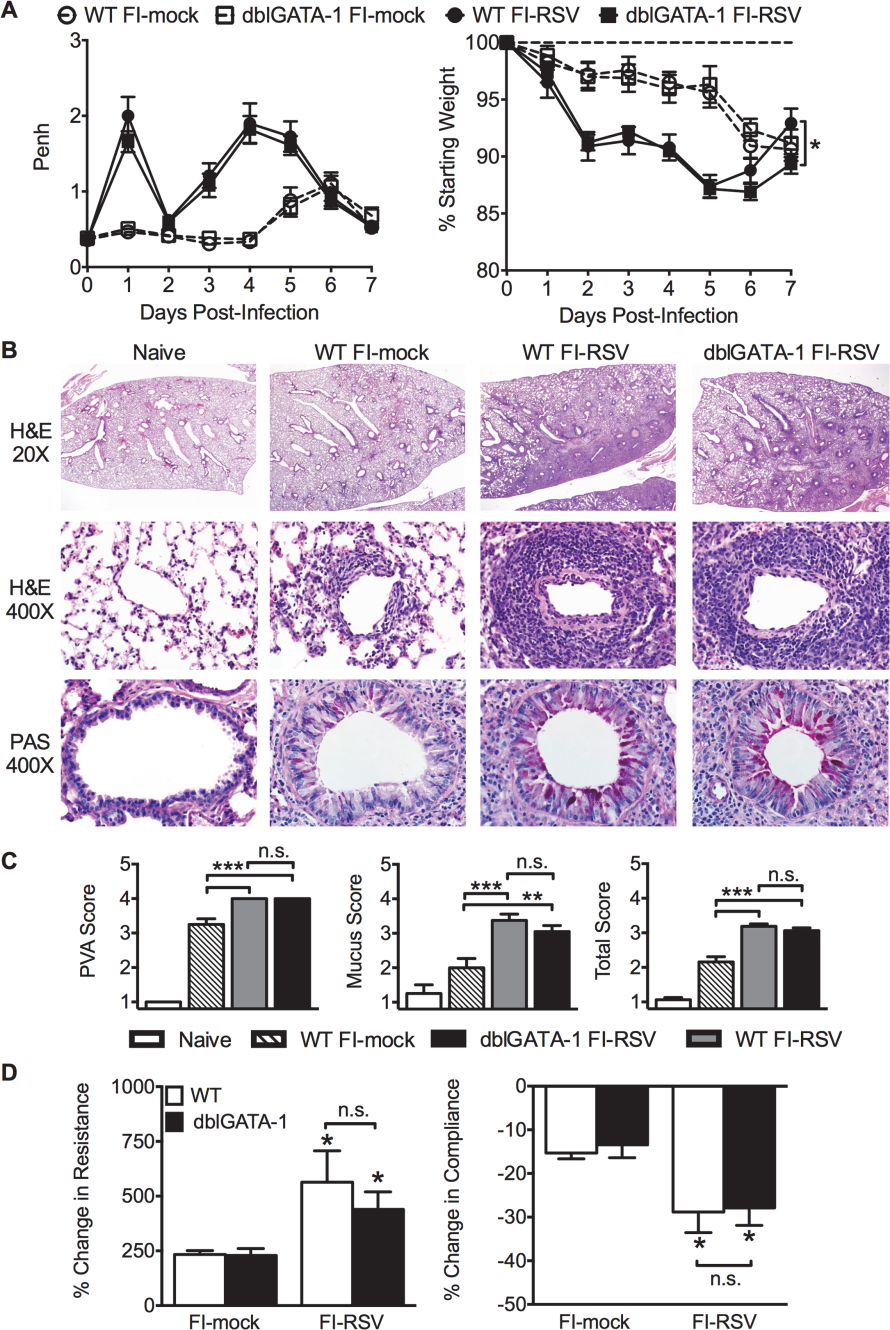
Eosinophils are not required to mediate FI-RSV VED. (A) WT and dblGATA-1 mice vaccinated with FI-RSV were assessed daily for airway obstruction and weight loss following RSV challenge. (B) Hematoxylin and eosin (H&E) and periodic acid-Schiff (PAS) staining were performed on lungs from immunized mice 4 days following RSV challenge. Numbers to the left indicate power of magnification. (C) Perivascular aggregates of leukocytes (PVA), mucus, and total histology scores from immunized WT and dblGATA-1 mice were determined on day 4 following infection. (D) Airway resistance and compliance were determined in vaccinated mice 4 days post-infection. Data is represented as percentage change following methacholine administration over baseline values obtained following saline treatment. Data are represented as mean ± SEM of two independent experiments (*n* = 12 mice total for A, *n* = 8 for B-D). Groups were compared using one-way ANOVA at each time point, * p<0.05, ** p<0.01, *** p<0.001.

Airway obstruction (Fig. 2A) was not significantly altered between eosinophil-deficient and wild-type (WT) FI-RSV-immunized mice following RSV challenge. Weight loss through day 6 following RSV challenge was also largely unaffected in FI-RSV-vaccinated dblGATA-1 mice. On day 7 post-infection, weight recovery was slightly delayed in FI-RSV-immunized eosinophil-deficient mice. We also compared the histopathology between vaccinated WT and eosinophil-deficient mice on day 4 following RSV challenge. Following RSV infection of mock-immunized mice, an increase in leukocytic aggregates around airways and mucus hypersecretion was noted as compared to naive mice (Fig. 2B and C). However, neither histopathology nor mucus levels were significantly altered in the absence of eosinophils in FI-RSV-immunized mice.

To further assess lower airway function, we evaluated airway hyperresponsiveness (AHR) during mechanical ventilation following a methacholine challenge of vaccinated mice. Airway resistance was significantly (p<0.05) increased and tissue compliance was significantly (p<0.05) reduced in FI-RSV-immunized mice following RSV challenge at day 4 post-infection (Fig. 2D). However, airway resistance and compliance were similar following RSV challenge of FI-RSV-vaccinated WT and eosinophil-deficient mice (Fig. 2D). Taken together, these data demonstrate that, in contrast to the current prevailing notion, eosinophils are not required to mediate any of the characteristic disease parameters that are associated with FI-RSV VED.

### Th2-associated immune responses are crucial for the induction of AHR

**Fig 3:**
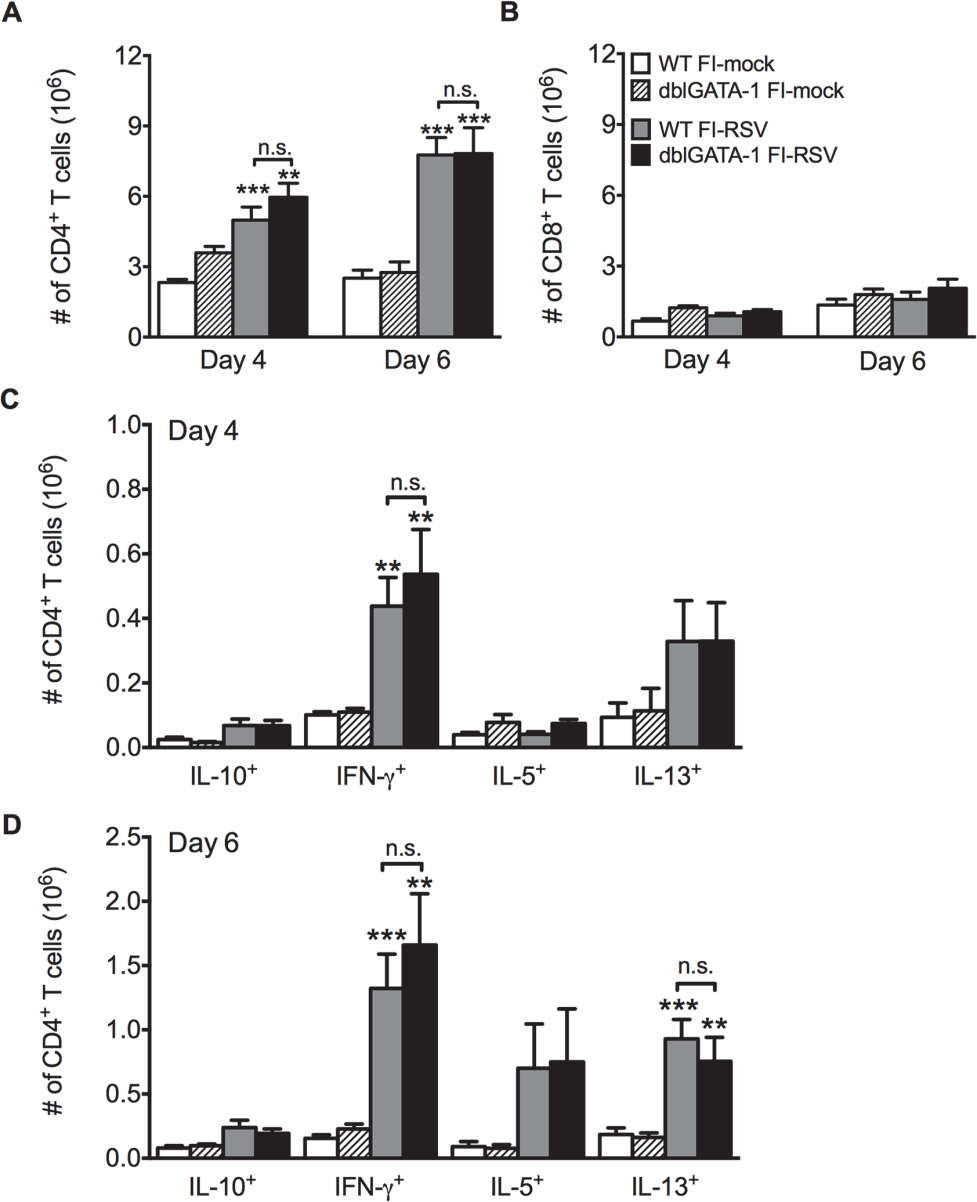
Absence of eosinophils does not affect T cell responses. Total number of (A) CD4 and (B) CD8 T cells in the lung of vaccinated WT and eosinophil-deficient mice was enumerated on days 4 and 6 following RSV infection. Number of IL-10, IFN-γ, IL-5, and IL-13 producing CD4 T cells in the lungs of immunized WT and dblGATA-1 mice was determined on days (C) 4 and (D) 6 post-infection. Data are represented as mean ± SEM of two independent experiments (*n* = 7 mice total for FI-mock, *n* = 8 mice for FI-RSV). Groups were compared using one-way ANOVA, ** p<0.01, *** p<0.001.

Previous work has shown that antibody-mediated depletion of CD4 T cells in FI-RSV vaccinated mice prior to RSV challenge ameliorates pulmonary histopathology suggesting a vital role of CD4 T cells in mediating pulmonary inflammation following RSV challenge [20]. We observed a significant (p<0.001) increase in the number of CD4 T cells in the lung on days 4 and 6 post-RSV challenge (Fig. 3A) of FI-RSV-immunized mice compared to the mock-immunized control group. Importantly, the number of CD4 T cells in the lung was not significantly altered in the absence of eosinophils (Fig. 3A). Consistent with the notion that inactivated vaccines are poor at eliciting CD8 T cell responses, we have previously reported that FI-RSV immunization fails to induce an RSV-specific CD8 T cell memory response [21,22]. In agreement with our previous results, we observed no significant increase in the CD8 T cell response of FI-RSV-vaccinated mice (Fig. 3B). In contrast, we observed a robust secondary CD4 T cell response. We next evaluated subsets of CD4 T cells by intracellular cytokine staining following PMA and ionomycin restimulation (S2 Fig.). By day 6 following RSV challenge there was a significant (p<0.01) increase in the number of CD4 T cells that produced either IFN-γ or IL-13 following restimulation in FI-RSV-immunized mice as compared to mock control groups regardless of whether or not eosinophils were present (Fig. 3C and D). Our results indicate that independent of the presence of eosinophils, FI-RSV immunization primes both a Th1 and Th2 memory CD4 T cell response that may promote disease associated with FI-RSV VED.

**Fig 4:**
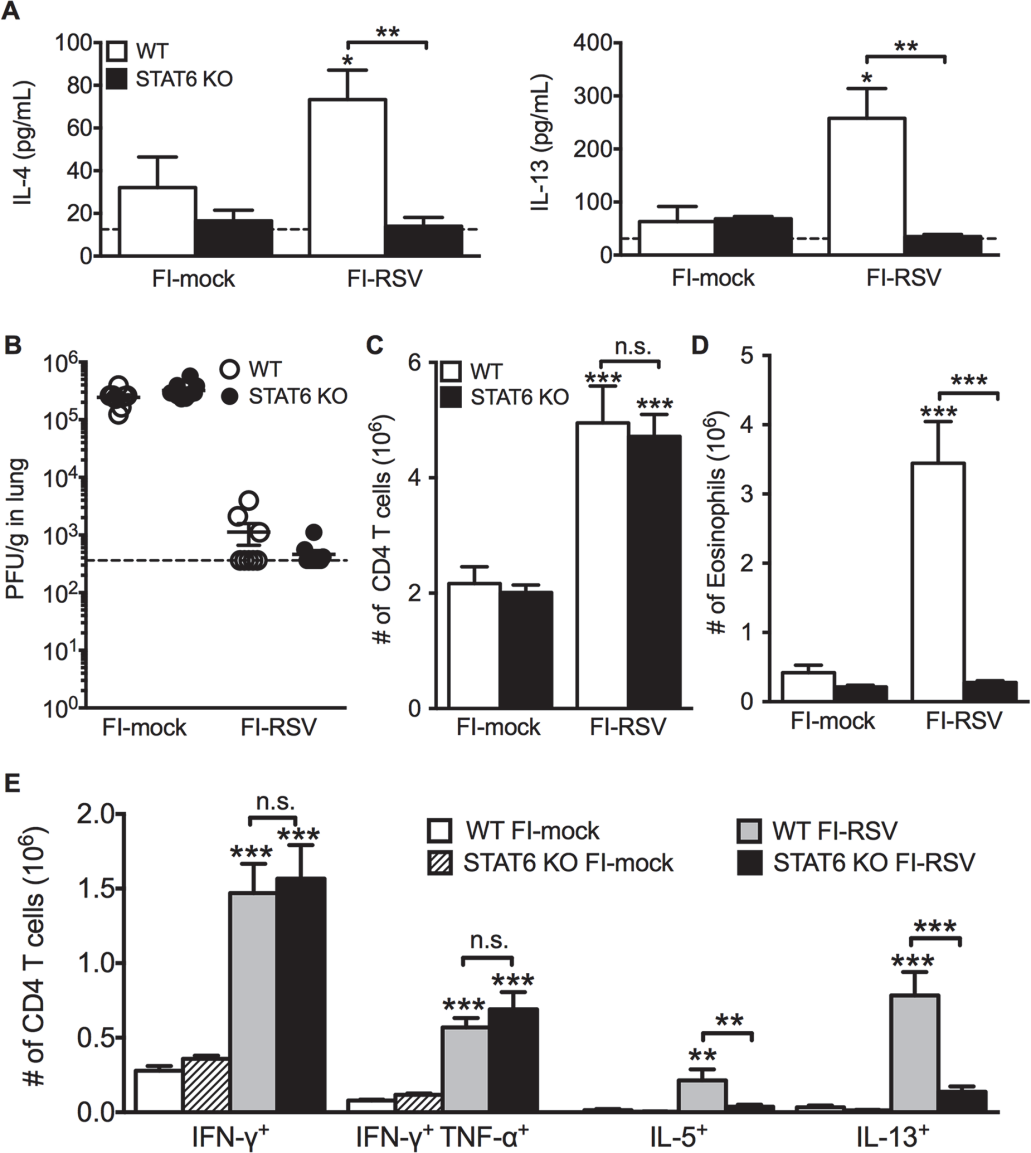
Th2-associated immune response is impaired in STAT6-deficient mice. (A) Cytokine protein amounts in the lungs of WT and STAT6 KO immunized mice were assessed on day 3 post-infection. Naïve controls were below the limit of detection for each cytokine. (B) RSV titers in the lungs of immunized WT and STAT6-deficient mice were determined via plaque assay on day 4 following RSV challenge. Number of (C) CD4 T cells and (D) eosinophils in the lungs of immunized mice were quantified on day 7 post-infection. (E) Number of Th1 and Th2 cytokine producing CD4 T cells in vaccinated WT and STAT6 KO mice on day 7 following RSV challenge were determined following PMA and ionomycin stimulation. Limit of detection for cytokine ELISA and plaque assay is denoted as a dotted line. Data are represented as mean ± SEM of two independent experiments (*n* = 8 mice total for A, B, *n* = 12 mice total for C-E). Groups were compared using one-way ANOVA at each time point, * p<0.05, ** p<0.01, *** p<0.001.

We next questioned if Th2-associated responses were responsible for mediating all disease parameters associated with FI-RSV VED. The transcription factor STAT6 is crucial for the differentiation of naive CD4 T cells to Th2 cells and ultimately the induction of Th2-associated immune responses [23]. Therefore, we utilized STAT6-deficient mice to determine if Th2-biased immune responses were necessary to mediate all disease symptoms associated with FI-RSV VED. FI-RSV-immunized mice on day 3 post-infection exhibited a significant (p<0.01) reduction in both IL-4 and IL-13 protein amounts in the lung (Fig. 4A). STAT6 deficiency did not impact lung viral titers in either mock- or FI-RSV-immunized mice (Fig. 4B). The total number of CD4 T cells remained similar between WT and STAT6-deficient FI-RSV-immunized mice (Fig. 4C). However, on day 7 following RSV challenge FI-RSV-immunized STAT6-deficient mice exhibited a significant (p<0.001) reduction in the number of eosinophils in the lung (Fig. 4D). Moreover, the number of IL-5- and IL-13-producing CD4 T cells was significantly (p<0.01) reduced in FI-RSV-immunized STAT6-deficient mice at day 7 post-infection (Figs. 4E and S3). However, the number of IFN-γ-producing CD4 T cells remained similar in the lungs between FI-RSV-immunized STAT6-deficient and WT mice (Fig. 4E). These data demonstrate that the overall Th2-associated immune response in STAT6-deficient mice is severely diminished.

**Fig 5:**
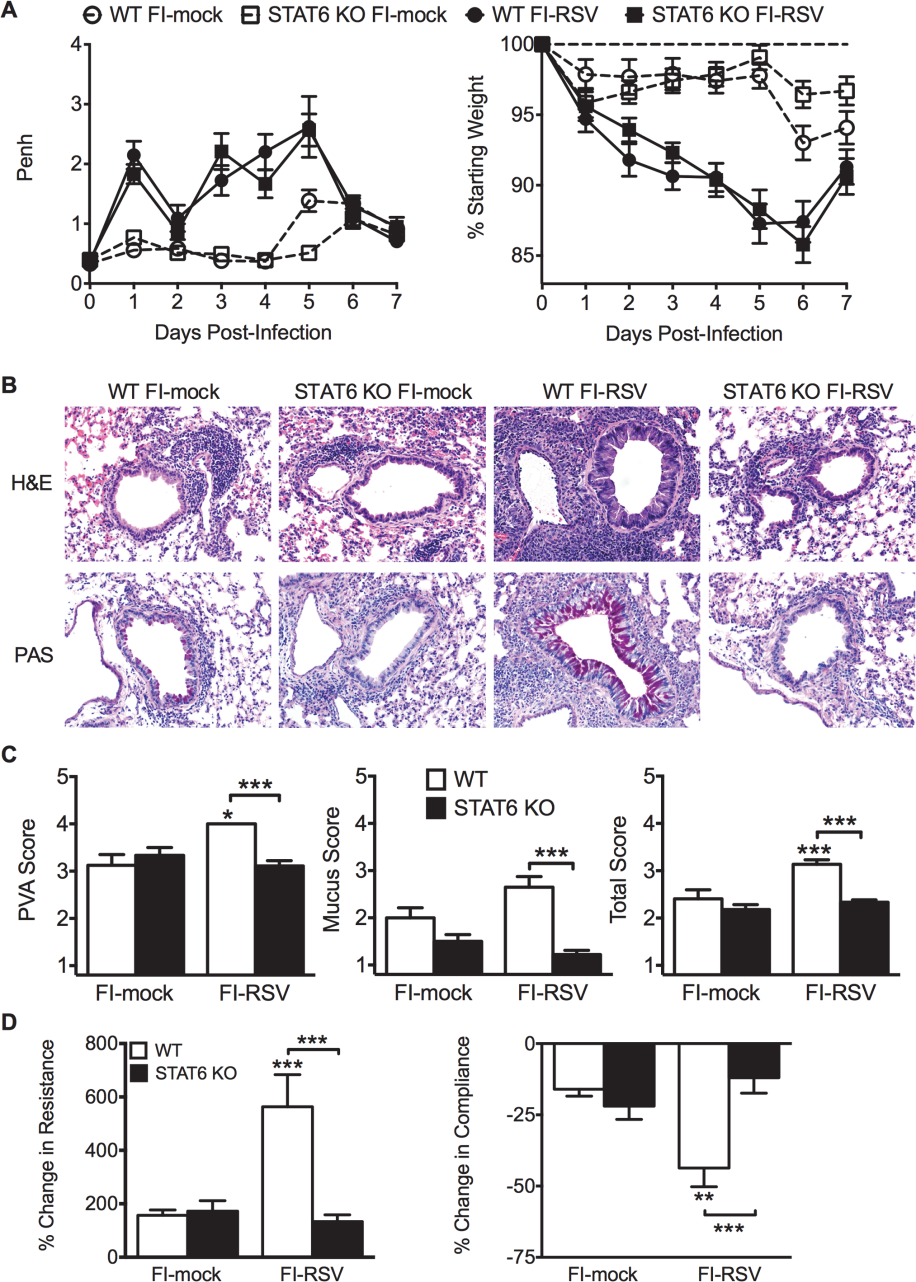
Th2-associated immune response is necessary for lung pathology and airway hyperreactivity. (A) Mock- or FI-RSV-immunized WT and STAT6-deficient mice were assessed daily for airway obstruction and weight loss following RSV challenge. (B) H&E and PAS staining on lung sections from immunized WT and STAT6 KO mice was performed on day 4 following RSV infection. Representative pictures for each group were taken at 200X magnification. (C) Stained lung sections were scored for degree of PVA, mucus, and total histopathology score. (D) Airway resistance and compliance in vaccinated WT or STAT6-deficient mice was evaluated on day 4 post-infection. Data are represented as mean ± SEM of two independent experiments (*n* = 12 mice total for A, *n* = 8 total mice for B-D). Groups were compared using one-way ANOVA at each time point, * p<0.05, ** p<0.01, *** p<0.001.

We next sought to determine if the impaired Th2-associated immune response in STAT6-deficient mice would ameliorate disease associated with FI-RSV VED. The absence of STAT6-signaling did not significantly alter either the airway obstruction or weight loss (Fig. 5A) in FI-RSV-immunized mice following RSV challenge. In contrast, assessment of histopathology revealed multiple changes to the lung environment (Figs. 5B and S4). Specifically, FI-RSV-immunized STAT6-deficient mice exhibited significantly (p<0.001) reduced perivascular leukocytic aggregates as compared to the WT group (Fig. 5C). Mucus hypersecretion was also significantly (p<0.001) reduced in STAT6-deficient FI-RSV-immunized mice as compared to the WT control group. Assessment of AHR also revealed a significant (p<0.001) reduction in airway resistance and a significant (p<0.001) improvement in compliance (Fig. 5D) of FI-RSV-immunized STAT6-deficient mice as compared to WT control group. Overall, our results demonstrate that the Th2-associated immune response does not substantially contribute to either airway obstruction or weight loss associated with FI-RSV VED. However, the Th2-associated immune response is required to induce lower airway pathology, mucus hypersecretion and AHR.

### TNF-α contributes to both airway obstruction and weight loss associated with FI-RSV VED

**Fig 6:**
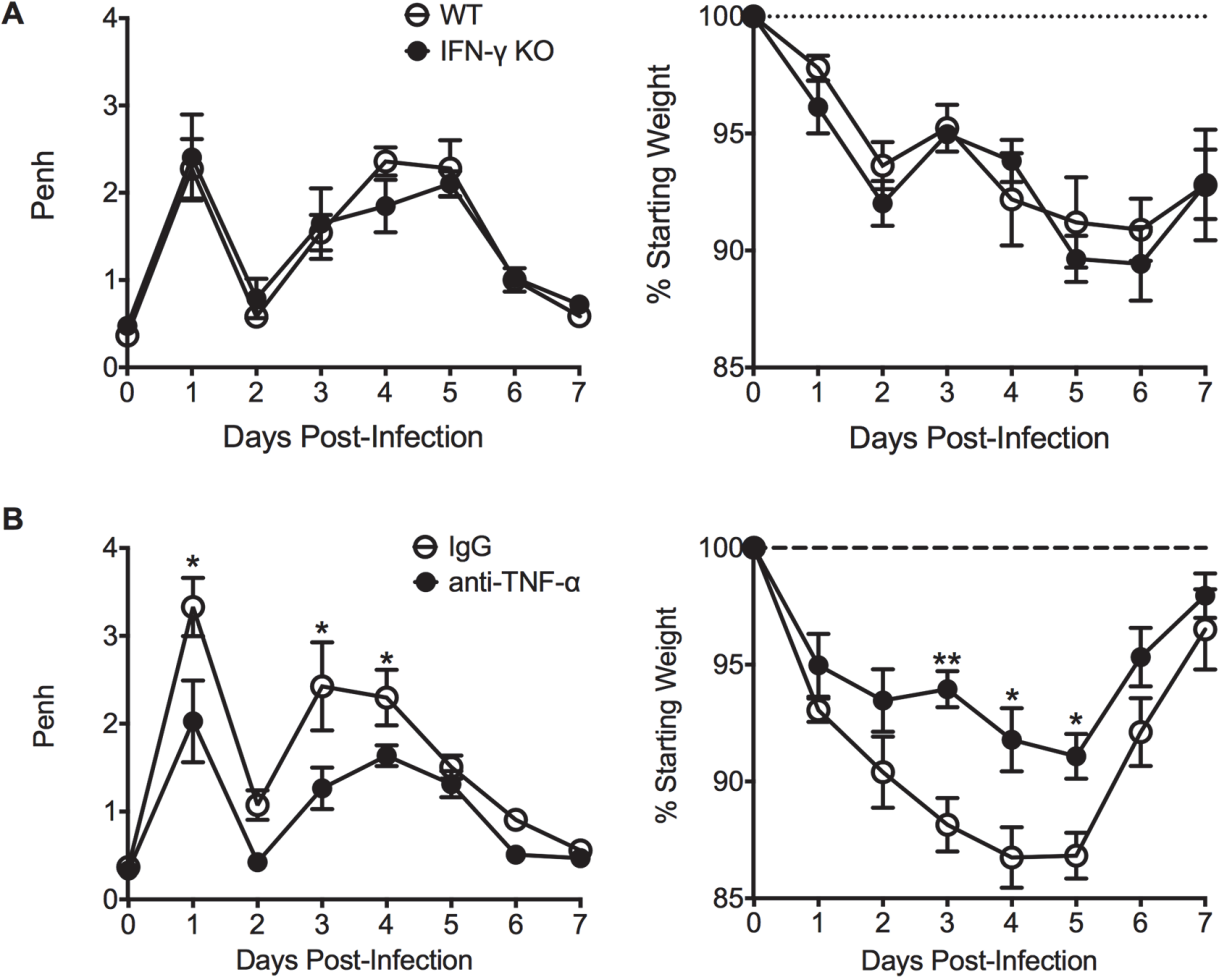
TNF-α contributes to airway obstruction and weight loss during FI-RSV VED. (A) WT and IFN-γ-deficient mice immunized with FI-RSV were challenged with RSV 3 weeks later and airway obstruction and weight loss were assessed daily. (B) BALB/c mice were immunized with FI-RSV and treated with either IgG or anti-TNF-α neutralization antibody prior to RSV challenge. Mice were assessed for airway obstruction and weight loss daily following infection. Data are represented as mean ± SEM of 2 independent experiments (*n* = 8 total mice). Groups were compared using one-way ANOVA, * p<0.05, ** p<0.01, *** p<0.001.

We next investigated other immunological factors related to the CD4 T cell response that could mediate either the severe weight loss or airway obstruction associated with FI-RSV VED. Since there is a significant (p<0.01) increase the number of IFN-γ- and TNF-α-producing CD4 T cells in FI-RSV-immunized mice following RSV infection (Figs. 3C and D, and 4E), we assessed the roles of IFN-γ and TNF-α during FI-RSV VED. Despite a significant increase in IFN-γ protein amounts in the lung at day 3 post-infection of FI-RSV-vaccinated mice (Fig. 1D), FI-RSV-immunized IFN-γ-deficient mice displayed no alteration in either airway obstruction or weight loss (Fig. 6A) as compared to WT controls. In contrast, neutralization of TNF-α in FI-RSV-immunized mice significantly (p<0.05) reduced both the increase in airway obstruction and weight loss (Fig. 6B) following RSV infection.

**Fig 7:**
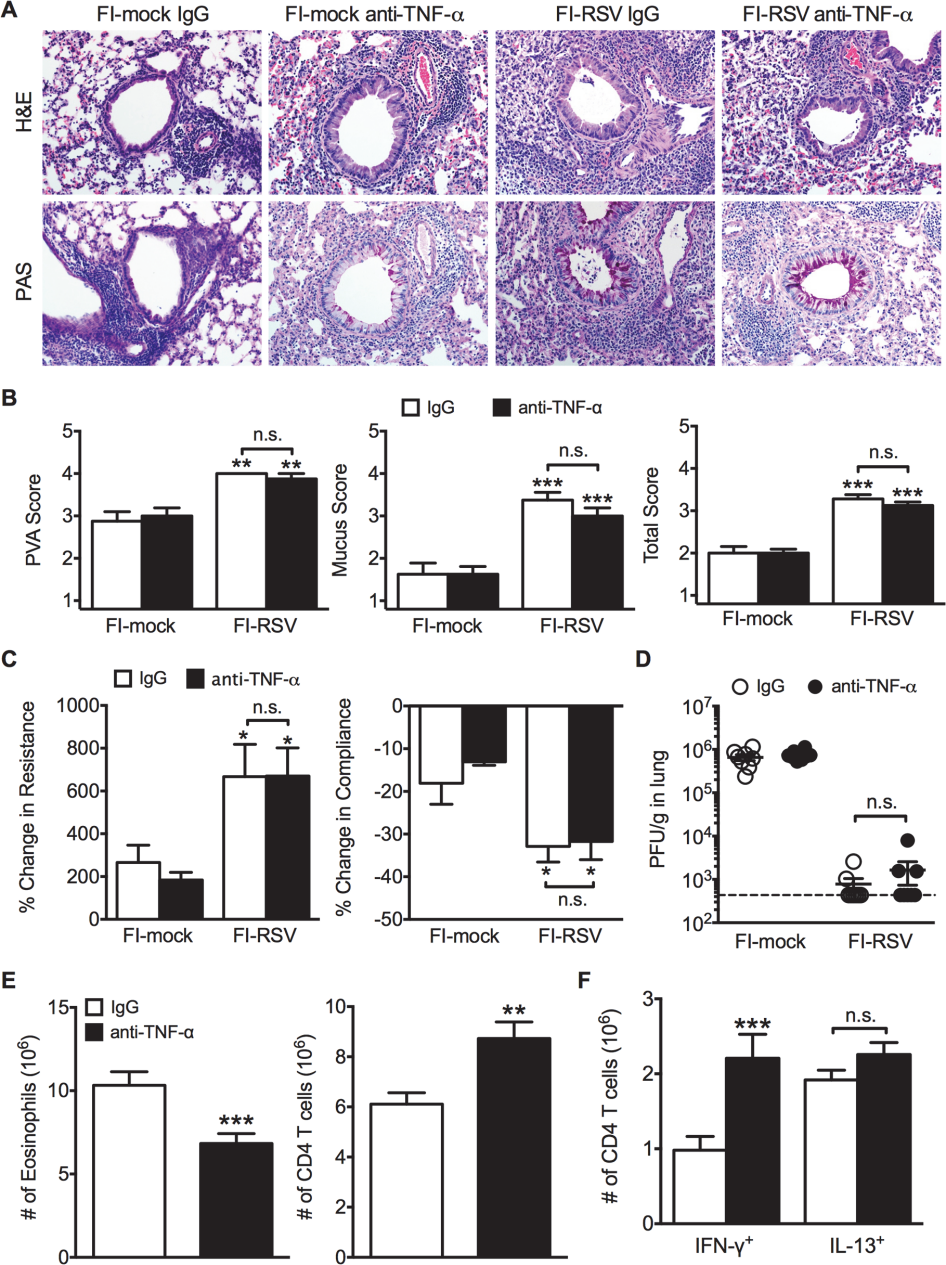
TNF-α contributes to airway obstruction and weight loss during FI-RSV VED. BALB/c mice were immunized with FI-RSV and treated with either IgG or anti-TNF-α neutralization antibody prior to RSV challenge. (A) H&E and PAS staining were performed on immunized mice at day 4 following RSV challenge. Representative pictures were taken for each group at 200X magnification. (B) Stained lung sections were evaluated for PVA, mucus, and total histopathology scores. (C) Lower airway resistance and compliance were assessed in immunized mice at day 4 p.i. (D) Viral titers were quantified at day 4 p.i. between IgG and anti-TNF-α treated immunized mice. (E) Total numbers of eosinophils and CD4 T cells in the lungs of vaccinated mice receiving anti-TNF-α neutralizing antibody were enumerated on day 7 following RSV infection. (F) Total number of Th1 and Th2 cytokine producing cells in the lungs was quantified at day 7 post-infection following PMA and ionomycin stimulation. Data are represented as mean ± SEM of 2 independent experiments (*n* = 8 total mice). Groups were compared using one-way ANOVA, * p<0.05, ** p<0.01, *** p<0.001.

To determine if TNF-α also contributed to both AHR and mucus hypersecretion, or if distinct immunological mechanisms mediate separate disease manifestations, we evaluated both histology and airway hyperreactivity. The scores for degree of perivascular leukocytic aggregates, mucus, and total histopathology were significantly (p<0.01, 0.001, and 0.001 respectively) increased in FI-RSV-immunized mice following TNF-α neutralization as compared to FI-mock-immunized controls (Fig. 7A and B). However, histopathology was similar in FI-RSV-immunized mice following either IgG or anti-TNF-α treatment (Fig. 7A and B). Furthermore, neutralization of TNF-α did not significantly alter either lower airway resistance or compliance following methacholine administration (Fig. 7C).

The similar histopathology and AHR in FI-RSV-immunized mice following TNF-α neutralization was not due to a difference in viral clearance as titers remained similar at day 4 p.i. (Fig. 7D). TNF-α neutralization led to a significant (p<0.001) decrease in the number of eosinophils (Fig. 7E) in the lung of FI-RSV-immunized mice. In addition, TNF-α neutralization caused a significant (p<0.001) increase in CD4 T cell numbers (Fig. 7E) that correlated to an increase in the number of IFN-γ-producing, but not IL-13-producing CD4 T cells (Fig. 7F). Moreover, FI-RSV-immunized mice deficient in STAT6 signaling exhibit a similar amount of TNF-α protein in the lungs following RSV challenge as compared to WT controls (S5 Fig.). Overall, these data suggest that distinct immunological mechanisms affect different disease manifestations. Specifically, the Th2-biased immune response mediates AHR and mucus hypersecretion while TNF-α contributes to both airway obstruction and weight loss.

### CD4 T cells are required to mediate disease associated with FI-RSV VED

**Fig 8:**
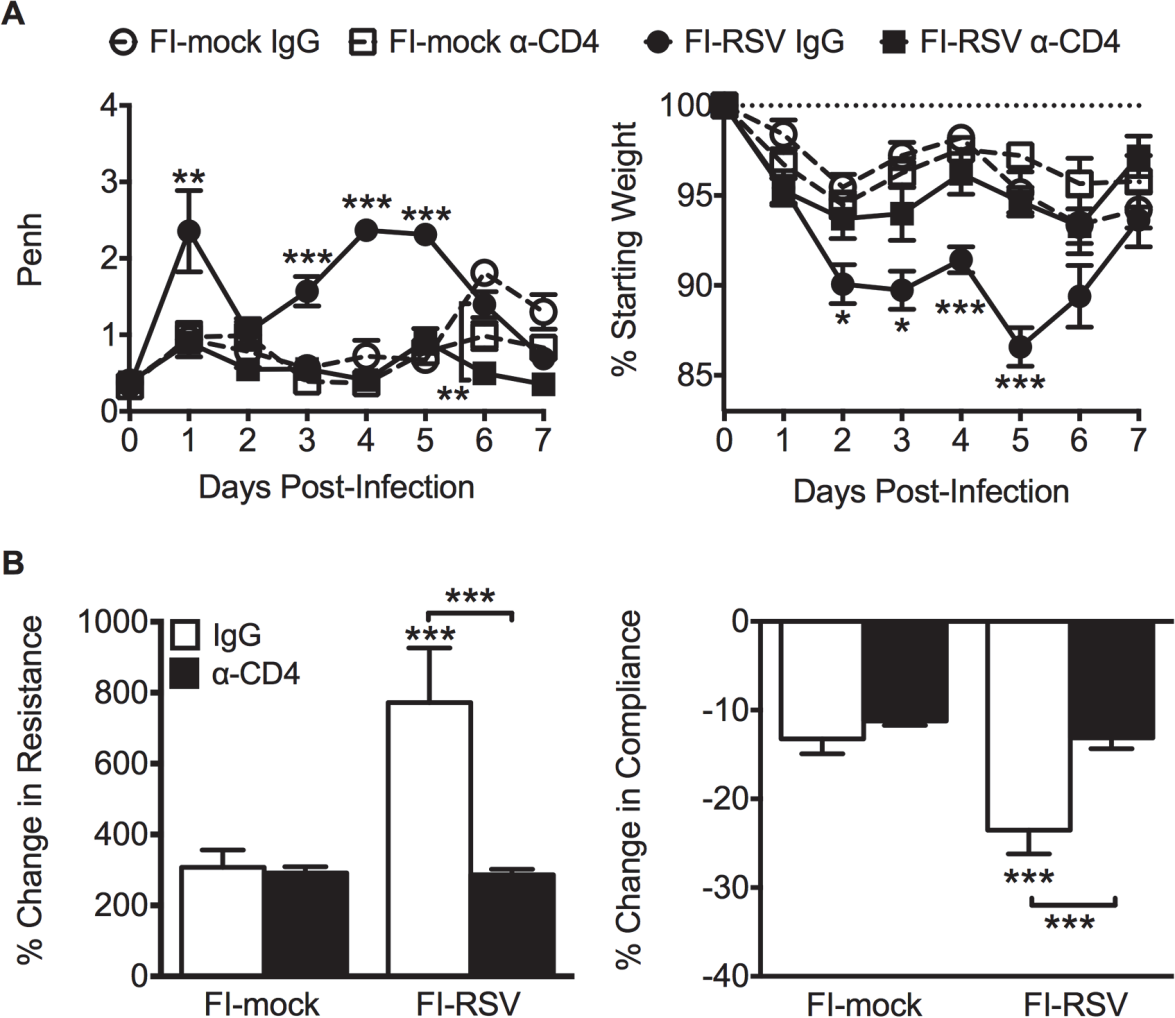
CD4 T cells are required to induce FI-RSV VED. (A) Airway obstruction and weight loss were determined daily following RSV challenge of either control or CD4 T cell depleted mice immunized with either FI-mock or FI-RSV. (B) Airway resistance and compliance were assessed in either control or CD4 T cell depleted immunized mice. Data are represented as mean ± SEM of 2 independent experiments (*n* = 8 mice total). Groups were compared using one-way ANOVA with Tukey-Kramer post-test analysis, * p<0.05, ** p<0.01, *** p<0.001.

**Fig 9:**
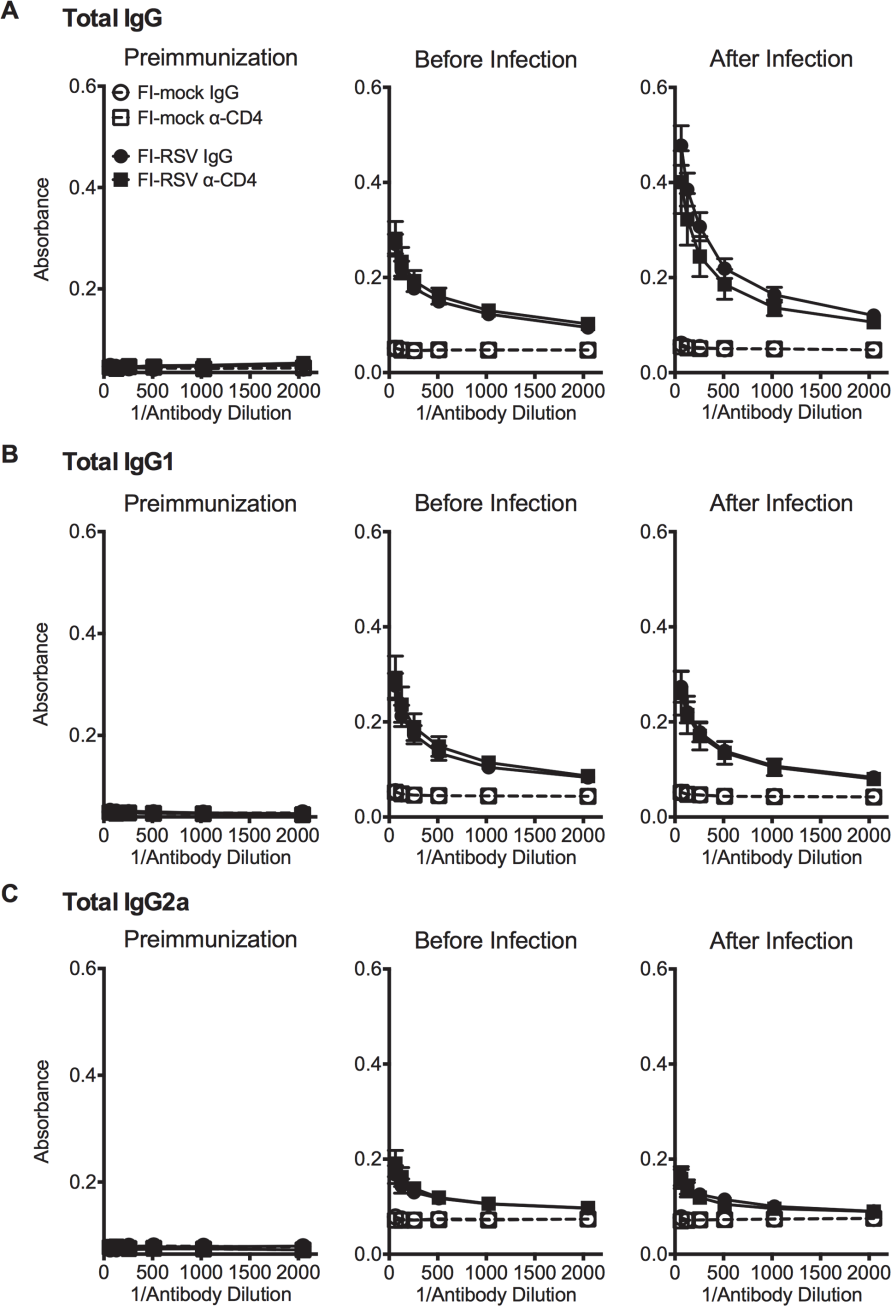
Antibody response is unaltered in CD4 T cell depleted mice. BALB/c mice were immunized with either FI-mock or FI-RSV, 21 days later treated i.p. with IgG or α-CD4 antibody, and challenged with RSV 2 days following antibody treatment. Serum was collected from mice preimmunization, before infection with antibody treatment, and after infection on day 4 post-infection. Serum was assessed for levels of (A) total IgG, (B) IgG1, and (C) IgG2a starting at a 1:64 dilution of serum. Data are represented as mean ± SEM of two independent experiments (*n* = 8 mice total). Groups were compared using one-way ANOVA with Tukey-Kramer post-test analysis.

Previous work has indicated that the induction of non-neutralizing antibody responses and immune complex deposition may contribute to the development of FI-RSV ERD [13,14]. In contrast, our data indicate that both Th1- and Th2-associated cytokines play a critical role in mediating individual disease parameters associated with FI-RSV VED. CD4 T cell responses are significantly (p<0.01) increased in FI-RSV-immunized mice following RSV challenge and consist of a mixture of Th1 and Th2 cells (Fig. 3C and D). In addition, it has been shown that CD4 T cells are required for the histopathology associated with FI-RSV VED [20]. Therefore, we sought to evaluate whether or not CD4 T cells were solely responsible in mediating pulmonary dysfunction and weight loss associated with FI-RSV VED. Depletion of CD4 T cells prior to RSV challenge, led to a significant (p<0.05) amelioration of both airway obstruction and weight loss (Fig. 8A) in FI-RSV-immunized mice. Furthermore, the absence of FI-RSV epitope-specific CD4 T cells significantly (p<0.001) reduced the changes in airway resistance and compliance (Fig. 8B) observed in FI-RSV-immunized mice after RSV challenge. Importantly, the amelioration of disease in FI-RSV-immunized mice occurred despite similar antibody levels between IgG- and α-CD4-treated groups for total IgG (Fig. 9A), IgG1 (Fig. 9B), and IgG2a (Fig. 9C). Our results show that CD4 T cells are necessary to orchestrate an immune response that mediates all facets of disease associated with FI-RSV VED.

## Discussion

The morbidity and mortality associated with the failed FI-RSV vaccine trial has significantly hampered the development of an RSV vaccine. Here we dissected the underlying immunological mechanisms that mediate individual disease parameters associated with FI-RSV VED. In contrast to the widely held belief that eosinophils play a critical role in the pathogenesis of FI-RSV VED [4], our results indicate that eosinophils are not required to mediate the crucial disease parameters associated with FI-RSV VED. This was an unexpected result given the more defined pathogenic role of eosinophils in other diseases such as hypereosinophilic syndrome and asthma [24–27]. Upon re-examination of the human autopsy specimens from the initial FI-RSV vaccine trials, Prince et al. found that only 1–2% of cells in the bronchial lumen and peribronchiolar region were eosinophils [12]. The vast majority of inflammatory cells in the bronchial epithelium were neutrophils and lymphocytes. Similar findings were observed in lung sections from FI-RSV-immunized cotton rats, another animal model that exhibits FI-RSV ERD [12]. However, one of the reports from the original FI-RSV vaccine trials also noted elevated numbers of eosinophils in the peripheral blood of 56% of the vaccine recipients indicating that eosinophilia was likely a common feature of the response [5]. Our vaccinated mice also develop pulmonary eosinophilia in greater numbers as compared to an acute infection setting following RSV challenge, but they do not exhibit an increase in neutrophil numbers. The number of neutrophils is significantly increased in the peripheral blood leukocytes of infants with a severe RSV infection [28]. It is unclear if the number of lung neutrophils in FI-RSV-immunized humans and cottons rats would be increased as compared to mock-immunized control groups. Thus, the potential role neutrophils may have played in FI-RSV VED remains unclear.

It has also been suggested that eosinophils may play a beneficial role during the innate immune response to aid in RSV clearance as observed in a hypereosinophilic, IL-5 transgenic mouse model [29]. However, we observed no significant alteration in viral titers during FI-RSV VED in eosinophil-deficient mice on either day 4 or 7 post RSV infection. These differences in viral clearance are likely due to the transgenic murine strain used by Phipps et al. in which there is constitutive production of IL-5 in the periphery. Therefore, these transgenic mice would have a supraphysiologic number of eosinophils in the lung prior to viral challenge. Our data indicate that eosinophils are recruited to the lung as a consequence of the increased levels of Th2-associated cytokines and chemotactic agents such as IL-5, IL-13, and eotaxin [30,31]. It is conceivable that eosinophils may play a beneficial role by contributing to airway remodeling of the lung tissue with minimal impact on respiratory function [32,33]. However, based on our results it is clear that other cell types are primarily responsible for mediating FI-RSV VED.

Our data demonstrate that a Th2-biased immune response is necessary to induce AHR and mucus hypersecretion. Th2 cells can mediate pulmonary inflammation during allergic responses through several methods including induction of mucus hypersecretion, indirectly promoting airway smooth muscle contraction, and mediating chemotaxis of other inflammatory cells such as eosinophils and mast cells [34–36]. Th2-biased immune responses play a critical role in orchestrating chronic disease manifestations associated with asthma including both pulmonary inflammation and bronchoconstriction [37]. However, while a Th2-biased immune response was essential to promote AHR and histopathology, it did not have a significant impact on airway obstruction and weight loss. The increased airway obstruction associated with FI-RSV immunization in the absence of STAT6-signaling suggests that baseline pulmonary function remains impaired despite reduced AHR. In contrast, the neutralization of TNF-α led to the significant reduction in airway obstruction and no alteration to AHR. TNF-α plays a critical role in numerous respiratory diseases including asthma, chronic obstructive pulmonary disease, acute lung injury, and acute respiratory distress syndrome primarily through the induction of a proinflammatory environment, but can also directly cause apoptosis of human bronchial epithelium [38,39]. The difference in baseline pulmonary function and AHR indicates that these parameters may represent two separate disease manifestations. In agreement, previous studies have shown that baseline measurements in pulmonary function often do not correlate with airway hyperreactivity in asthmatic patients [40–43]. Overall, this indicates that specific disease manifestations are regulated by distinct immunological mechanisms.

Multiple cytokines have been documented to promote weight loss during either cancer or chronic infections including IL-1, IL-6, IFN-γ and TNF-α [44,45]. Due to the increased number of CD4 T cells that produced IFN-γ and TNF-α even in the absence of eosinophils or STAT6-signaling, we assessed the role of these cytokines in FI-RSV-immunized mice. Although IFN-γ protein amounts were significantly increased in FI-RSV-immunized mice, IFN-γ-deficient mice did not exhibit an alteration in either airway obstruction or weight loss suggesting a negligible role for IFN-γ in mediating disease. However, the neutralization of TNF-α led to a significant reduction in both airway obstruction and weight loss in FI-RSV-immunized mice. This is in agreement with previous work showing that prolonged TNF-α production promotes weight loss during an acute RSV infection [46]. As weight loss was not completely abolished in FI-RSV-immunized mice, this suggests that other proinflammatory cytokines are induced following RSV infection [47], such as IL-1 or IL-6, that may contribute to the weight loss associated with FI-RSV VED.

Our results show that CD4 T cells are necessary to mediate all disease parameters associated with FI-RSV VED including airway obstruction, weight loss, and AHR. The depletion of CD4 T cells led to a significant amelioration of all disease parameters as we hypothesized due to the above defined roles of both the Th1- and Th2-associated immune response in mediating distinct disease manifestations. In agreement with our results, antibody-mediated depletion of CD4 T cells [20] or antibody-mediated neutralization of the Th2-associated cytokine IL-4 in conjunction with neutralization of IL-10 [48] was previously shown to result in decreased histopathology in FI-RSV-immunized mice following RSV challenge. Taken together, these data indicate that the CD4 T cell response is critical to mediate the increased disease severity associated with RSV VED.

Our data illustrates that individual disease parameters are mediated by distinct subsets of CD4 T cells. The demonstration that Th2-associated cytokines as well as TNF-α production by Th1 cells drives the induction of FI-RSV VED should serve as areas of focus for evaluation of new RSV vaccine candidates for disease potentiation. Overall our results highlight the necessity to evaluate future RSV vaccine candidates by the careful examination of several disease parameters. Evaluation of only one or two disease parameters such as eosinophilia, histopathology or AHR, may overlook disease exacerbation in terms of weight loss or airway obstruction that is mediated by a distinct subset of memory CD4 T cells. Such actions could lead to undesirable results in vaccine trials and hamper further RSV vaccine development. Our results should provide a framework to thoroughly assess the safety of future RSV vaccines through the careful evaluation of critical disease parameters most associated with RSV VED.

## Materials and Methods

### Mice

Female BALB/cAnNCr mice between 6–8 wk old were purchased from the National Cancer Institute (Frederick, MD). Eosinophil-deficient dblGATA-1 (C.Cg-*Gata1* ^*tm6Sho*^/J) [19] and STAT6-deficient mice (C.129S2-*Stat6* ^*tm1Gru*^/J) on the BALB/c background were purchased from The Jackson Laboratory (Bar Harbor, ME). IFN-γ-deficient mice (C.129S7(B6)-Ifng^tm1Ts/J^) on the BALB/c background was obtained from John T. Harty (University of Iowa).

### Ethics statement

All experimental procedures utilizing mice were approved by the University of Iowa Animal Care and Use Committee. The experiments were performed under strict accordance to the Office of Laboratory Animal Welfare guidelines and the PHS Policy on Humane Care and Use of Laboratory Animals.

### Virus, immunization, and infection

The A2 strain of RSV was a gift from Barney S. Graham (National Institutes of Health, Bethesda, MD) and was propagated in HEp-2 cells (ATCC). Mice were infected intranasally (i.n.) with 1.5–1.7 x 10^6^ PFU RSV. FI-RSV was prepared from RSV infected Vero cells grown in OptiPRO^TM^ SFM media (Invitrogen). Growth of RSV in Vero cells produces virions that express a truncated form of the G protein lacking the C-terminus [49]. However, it has been previously shown that mice immunized with FI-RSV produced from a recombinant RSV lacking the entire G protein grown in Vero cells still exhibit FI-RSV vaccine-enhanced disease following RSV challenge [50]. Virus-infected cells were removed by scraping and sonicated for eight 1-sec pulses and subsequently centrifuged at 10,000 rpm for 10 min. Supernatant was inactivated with 10% formalin at 1:400 dilution for 72 hr at 37°C followed by centrifugation at 50,000 x g for 1 hr at 4°C. The pellet was resuspended in OptiPRO SFM at 1:25 dilution with 4 mg/mL of Imject Alum adjuvant (Thermo Fisher Scientific). The solution was centrifuged at 1000 x g for 30 min at 4°C and pellet was resuspended at 1:4 dilution in OptiPRO^TM^ SFM. The FI-RSV prep was sonicated in water bath sonicator for 15 sec on ice and stored in amber glass vials at 4°C. A mock preparation was also created using the same protocol from a lysate of Vero cells mock infected with PBS. Mice were vaccinated intramuscularly (i.m.) with 100 μl of a 1/200 dilution of FI-mock or FI-RSV in the lower right-hind flank. Mice were challenged with RSV 21 days following immunization.

### In vivo antibody depletion/neutralization

For CD4 T cell depletions, mice were treated with 250 μg of α-CD4 (clone GK1.5) antibody every 4 days i.p. starting at day -2 prior to RSV challenge. CD4 T cell frequencies were assessed in the peripheral blood leukocytes on day 0 prior to infection (anti-CD4 clone RM4–4) and found to be >99% depleted. For TNF-α neutralization, mice were administered 200 μg of anti-TNF-α (clone MP6-XT22) antibody both i.n. and i.p. at day -1 prior to infection. Every other day thereafter, mice were treated with an additional 200 μg dose of anti-TNF-α antibody i.p. As controls, mice were given a matching dose of control IgG antibody at similar timepoints and route of administration.

### Pulmonary function assessment

The lung function of mice was evaluated utilizing two methods of unrestrained whole-body plethysmography and forced oscillations via mechanical ventilation [51]. Enhanced pause (Penh) was measured using a whole-body plethysmograph (Buxco Electronics, Wilmington, NC) as previously described [52]. Penh was calculated based on pressure and volume changes over 5 min. While Penh is not a surrogate for lower airway resistance, it can be correlated to changes in baseline respiratory patterns [15,16,53]. Penh can also be used as an indication of airway obstruction and has been validated previously [17,18]. To investigate changes in lower airway hyperreactivity, mice were assessed on day 4 post-infection using a flexiVent mechanical ventilator (Scireq, Plattsburgh, NY). Mice were anesthetized with 100 mg/kg dose of pentabarbital and tracheotomized using a blunted 18 gauge needle. Respiratory mechanics were measured using the forced oscillation technique following saline and 25 mg/mL methacholine challenges administered using Aeroneb nebulizer. Methacholine is a bronchoconstrictor agent that induces airway constriction. Therefore, mice that exhibit greater airway reactivity will experience increased changes to parameters of pulmonary function, i.e. airway resistance and compliance, following methacholine challenge. Significant alterations in respiratory mechanics have been observed previously in murine models with RSV infection [54–57]. Airway resistance and compliance were expressed as percentage change over baseline measurements from saline treatment.

### Flow cytometry analysis

Lung and bronchoalveolar lavage (BAL) were harvested from mice as previously described [52,58]. Lung homogenates and BAL cells were surface-stained with mAbs specific to CD11c (clone N418), Siglec F (BD Biosciences, clone E50-2440), F4/80 (clone BM8), MHCII (clone M5/114.15.2), Ly6c (clone HK1.4), Ly6g (clone 1A8), CD90.2 (clone 53-2.1), CD4 (clone GK1.5) and CD8 (clone 53-6.7) for 30 min at 4°C and fixed with FACS lysing solution (BD Biosciences and eBioscience) for 10 min at room temperature. For intracellular cytokine staining (ICS), cells were stimulated for 5 hr at 37°C with 50 ng/mL PMA (Sigma-Aldrich) and 500 ng/mL ionomycin (Sigma-Aldrich) in the presence of 10 μg/mL brefeldin A (BFA, Sigma Aldrich) in 10% FCS-supplemented RPMI. Cells were then surface-stained for CD90.2 and CD4, fixed in FACS lysing solution, and stained intracellularly with mAbs specific to IFN-γ (clone XMG1.2), IL-10 (clone JES5-16E3), IL-17A (clone TC11-18H10.1), IL-5 (clone TRFK5), and IL-13 (eBioscience clone eBio13A) in FACS buffer containing 0.5% saponin (Sigma-Aldrich) for 30 min at 4°C. The total number of cytokine producing cells was calculated after subtraction of background staining using BFA only controls. All monoclonal antibodies were purchased from BioLegend unless otherwise stated. Stained cells were run on BD FACSCanto or LSRFortessa and analyzed with FlowJo (Tree Star, Ashland, OR) software.

### Antibody ELISA

Mouse serum was collected prior to immunization, 23 days following immunization with CD4 T cell depletion (2 days post-depletion), and 4 days following RSV challenge (CD4 depletion on days -2 and 2). Flat-bottom 96-well plates (Nunc MaxiSorp, Thermo Scientific) were coated with 1 x 10^4^ PFU/well of RSV overnight at 4°C. Plates were blocked with 5% non-fat dry milk in PBS for 2 hours at 37°C. Supernatants were serially diluted 1:2 starting at 1:64 over 6 total dilutions, and plates were incubated overnight at 4°C. RSV-specific antibody was detected using biotinylated goat anti-mouse antibody specific for IgG, IgG1, IgG2a, or IgE (Southern Biotech, Birmingham, AL) at a dilution of 1:500 for 2 hours at 37°C. Plates were incubated with 1:400 dilution of streptavidin-horse radish peroxidase conjugate (Sigma Aldrich) for 30 minutes at room temperature. Plates were developed in 0.1 mg/mL 3,3',5,5'-tetramethylbenzidine solution for 10 minutes and reaction was stopped with 2M sulfuric acid. Absorbance values (560 nm) were measured and assessed using Gen5 software (BioTek, Winooski, VT).

### Cytokine ELISA

Lungs were prepared for cytokine analysis as previously described [52,59]. Supernatants were analyzed for cytokines levels of IL-4 (eBioscience), IL-13 (R&D Systems, purified clone 38213.11 and biotinylated polyclonal goat anti-mouse IL-13), IL-17A (R&D Systems DuoSet ELISA Kit), and IFN-γ (eBioscience).

### Histology

Whole lungs were harvested on day 4 following RSV challenge and fixed in 10% neutral buffered formalin (Fischer Scientific). Lungs were processed as previously described [30] and stained for H&E for routine evaluation and PAS staining of amylase-treated tissue for mucus. Each sample was assessed for degree of interstitial disease, edema, perivascular aggregates of leukocytes (PVA), mucus, and a total score, a composite average of all disease parameters. Tissues were examined and scored in a manner masked to experiment groups [60]. Histopathologic scoring was similar to that previously described [52] and based on an ordinal scale in which a score “1” represented within a normal or naive range whereas a score of “4” represented extensive or severe processes. Specifically, the scoring definitions are as follows: PVA; 1—normal, within naïve parameters, 2—focal to uncommon numbers of solitary cells with uncommon aggregates, 3—multifocal moderate aggregates, 4—moderate to high cellularity and multifocal, large cellular aggregates that may be expansive into adjacent tissues, mucus; 1—no mucus, 2—epithelial mucinous hyperplasia with none to rare luminal mucus, 3—epithelial mucinous hyperplasia with luminal mucus accumulation in airways, 4—severe mucinous alterations, some airways may be completely obstructed by mucus.

### Plaque assays

Lung viral titers were determined as previously described [22,59]. Briefly, whole lungs were harvested from infected mice 4 days following infection, weighed, mechanically homogenized, and supernatant was stored at −80°C until further use. 1:10 serial dilutions of supernatants were performed and incubated on Vero cells (ATCC) in 6-well plates for 90 minutes at 37° C. Plates were rocked every 15 minutes and overlaid with a 1:1 mixture of Eagle minimum essential medium (EMEM, Lonza, Walkersville, MD) and 1% SeaKem ME agarose (Cambrex, North Brunswick, NJ). Following 5 days of incubation at 37° C, 5% CO_2_, plates were stained with a 1:1 mixture of EMEM and 1% agarose containing 1% neutral red (Sigma-Aldrich). Plaques were counted after 24–48 hours.

### Statistical analysis

All statistical analyses were performed using Prism software (GraphPad Software, San Diego, CA). Data was compared using unpaired, two-tailed Student *t* tests between two groups or one-way ANOVA with Tukey-Kramer post-test analyses for more than two groups, to determine if there was a statistical significance of at least α = 0.05. Asterisks are used to define a difference of statistical significance between the indicated group and its respective control group unless otherwise indicated by a line.

## Supporting Information

S1 Fig No significant alteration to Th17 response associated with FI-RSV immunization. (A) IL-17A protein amount in the lung was assessed at day 3 p.i. in FI-mock- and FI-RSV-immunized mice via ELISA. At day 4 p.i., lung cells were incubated with BFA and stimulated with PMA and ionomycin. (B) Number of IL-17A-producing CD4 T cells was evaluated in the lung. Data are represented as mean ± SEM of two independent experiments (*n* = 8 mice total). Groups were compared using Student’s t test.
(TIFF)

S2 Fig Gating strategy for cytokine staining on lungs of dblGATA-1 mice for flow cytometry. WT and dblGATA-1 mice were immunized with FI-RSV and challenged with RSV 21 days later. At day 4 p.i. lung cells were incubated with BFA and either left unstimulated or stimulated with PMA and ionomycin. Representative flow plots of (A) cytokines IL-10 and IFN-γ and (B) Th2 cytokines IL-5 and IL-13 for CD4 T cells at day 4 following RSV infection. Colored boxes indicate area used to quantify each cytokine. Samples were run on BD FACSCanto.
(TIFF)

S3 Fig Gating strategy for cytokine staining on lung cells from STAT6 KO mice. WT and STAT6 KO mice were immunized with FI-RSV and challenged with RSV 21 days later. At day 7 p.i. lung cells were incubated with BFA and either left unstimulated or stimulated with PMA and ionomycin. Representative flow plots of (A) Th1 cytokines IFN-γ and TNF-α and (B) Th2 cytokines IL-5 and IL-13 for CD4 T cells at day 4 following RSV infection. Colored boxes indicate area used to quantify each cytokine. Samples were analyzed on the BD LSRFortessa flow cytometer.
(TIFF)

S4 Fig Th2-associated immune response is required for histopathology and mucus hypersecretion. H&E staining on lung sections of immunized WT and STAT6 KO mice was performed on day 4 following RSV infection. H&E and PAS staining on lung sections from naïve mice. Representative pictures for each group were taken at the indicated magnifications.
(TIFF)

S5 Fig TNF-α protein amount is not altered in STAT6 KO FI-RSV-immunized mice. TNF-α protein amount in the lung was assessed at day 3 p.i. in WT and STAT6 KO FI-RSV-immunized mice via ELISA. Data are represented as mean ± SEM of two independent experiments (*n* = 8 mice total). Groups were compared using Student’s t test.
(TIFF)
