# Supplementary figures and images for: RSV Vaccine-Enhanced Disease Is Orchestrated by the Combined Actions of Distinct CD4 T Cell Subsets
Source: PLoS Pathog. 2015 Mar 13;11(3):e1004757. doi: 10.1371/journal.ppat.1004757 (PMC4358888; doi:10.1371/journal.ppat.1004757)

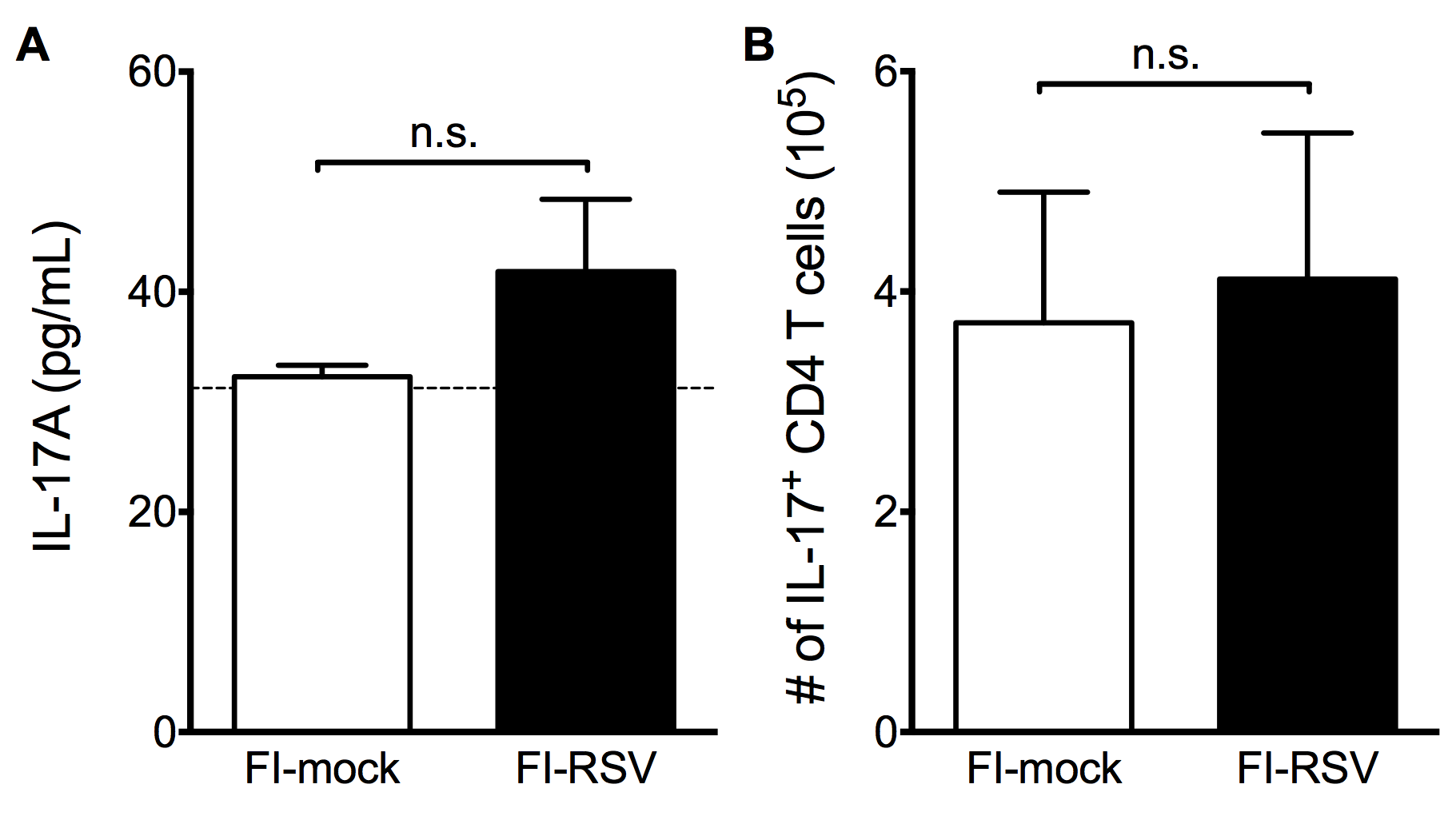

Supplement: S1 Fig — (A) IL-17A protein amount in the lung was assessed at day 3 p.i. in FI-mock- and FI-RSV-immunized mice via ELISA. At day 4 p.i., lung cells were incubated with BFA and stimulated with PMA and ionomycin. (B) Number of IL-17A-producing CD4 T cells was evaluated in the lung. Data are represented as mean ± SEM of two independent experiments (n = 8 mice total). Groups were compared using Student’s t test. (TIFF) [file ppat.1004757.s001.tiff]

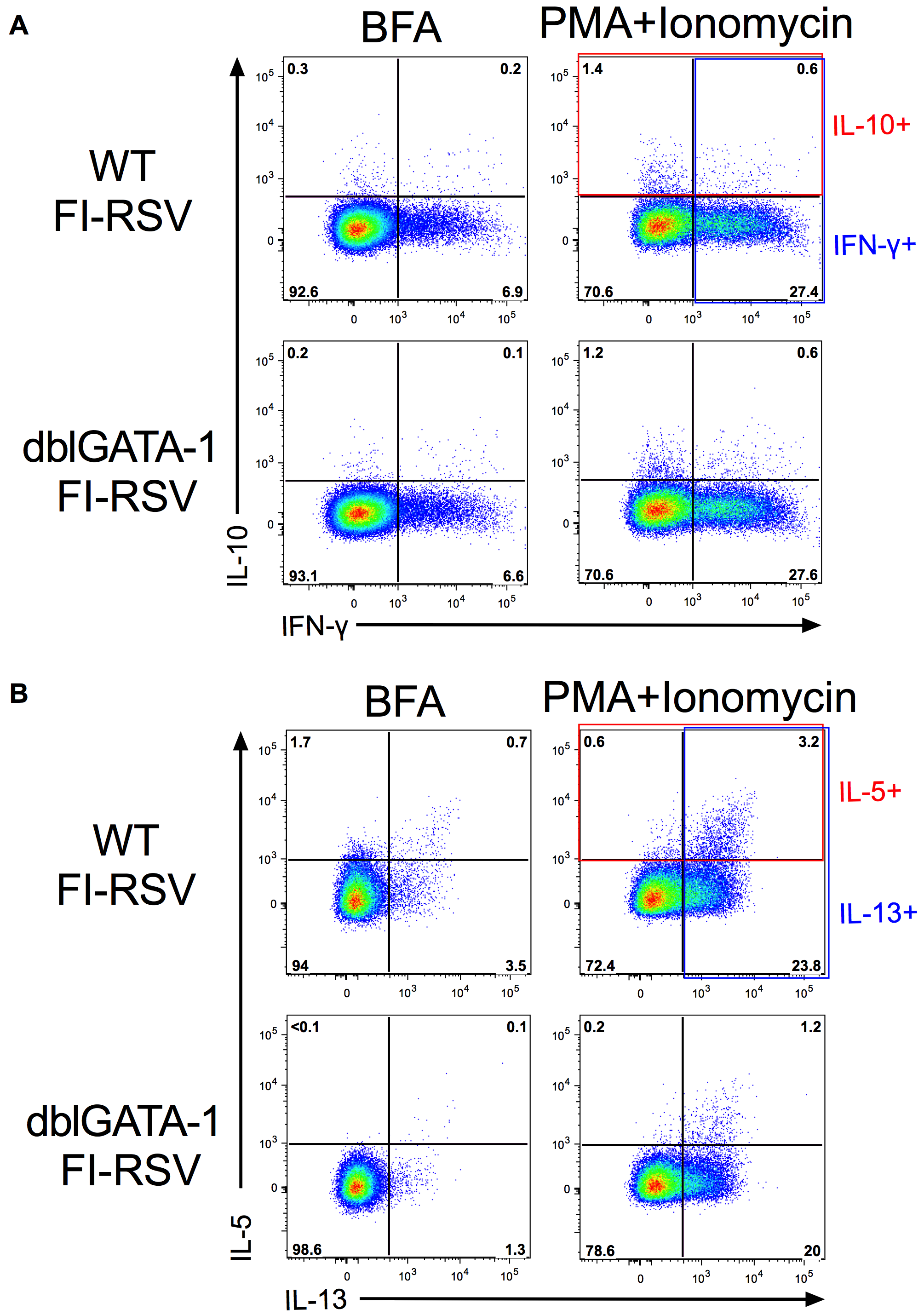

Supplement: S2 Fig — WT and dblGATA-1 mice were immunized with FI-RSV and challenged with RSV 21 days later. At day 4 p.i. lung cells were incubated with BFA and either left unstimulated or stimulated with PMA and ionomycin. Representative flow plots of (A) cytokines IL-10 and IFN-γ and (B) Th2 cytokines IL-5 and IL-13 for CD4 T cells at day 4 following RSV infection. Colored boxes indicate area used to quantify each cytokine. Samples were run on BD FACSCanto. (TIFF) [file ppat.1004757.s002.tiff]

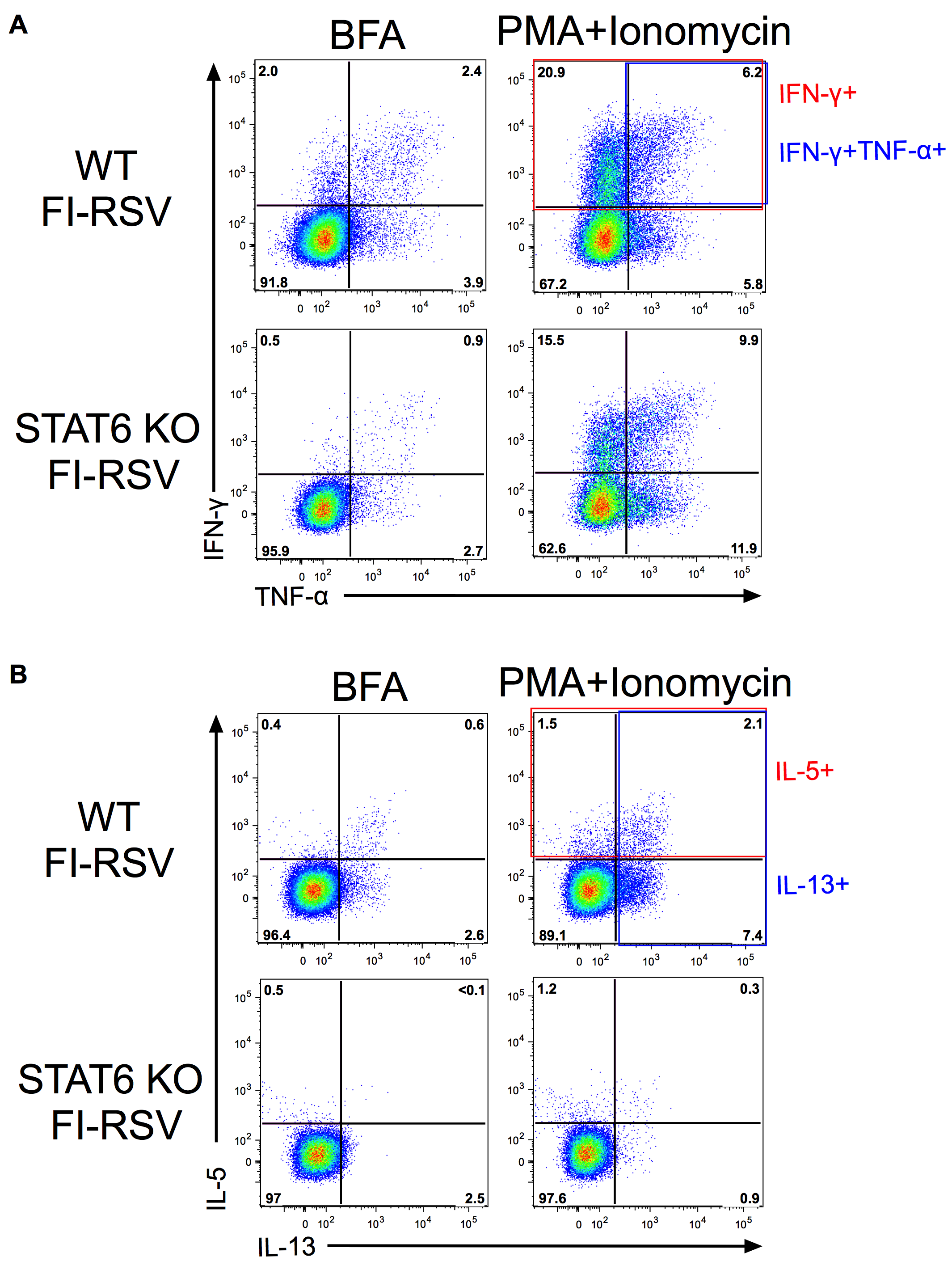

Supplement: S3 Fig — WT and STAT6 KO mice were immunized with FI-RSV and challenged with RSV 21 days later. At day 7 p.i. lung cells were incubated with BFA and either left unstimulated or stimulated with PMA and ionomycin. Representative flow plots of (A) Th1 cytokines IFN-γ and TNF-α and (B) Th2 cytokines IL-5 and IL-13 for CD4 T cells at day 4 following RSV infection. Colored boxes indicate area used to quantify each cytokine. Samples were analyzed on the BD LSRFortessa flow cytometer. (TIFF) [file ppat.1004757.s003.tiff]

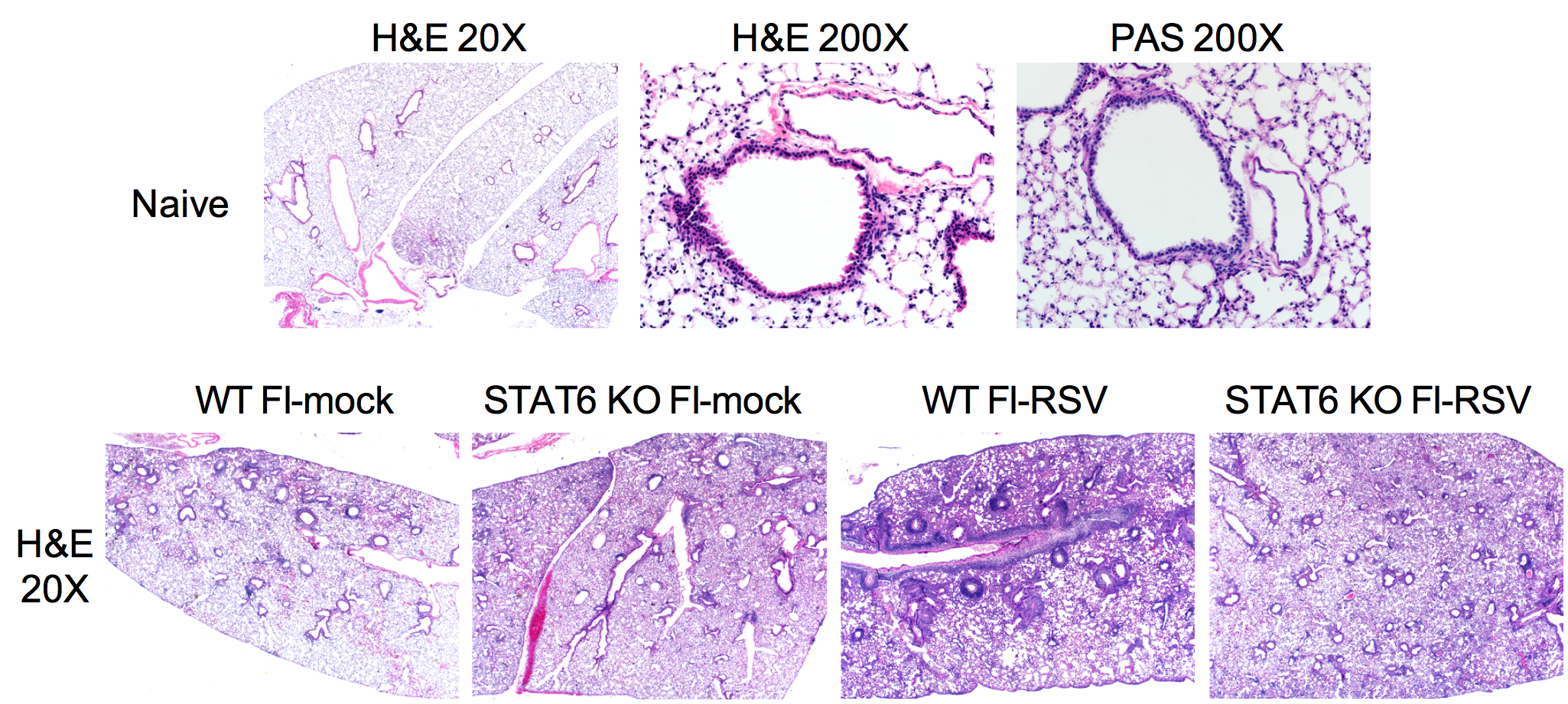

Supplement: S4 Fig — H&E staining on lung sections of immunized WT and STAT6 KO mice was performed on day 4 following RSV infection. H&E and PAS staining on lung sections from naïve mice. Representative pictures for each group were taken at the indicated magnifications. (TIFF) [file ppat.1004757.s004.tiff]

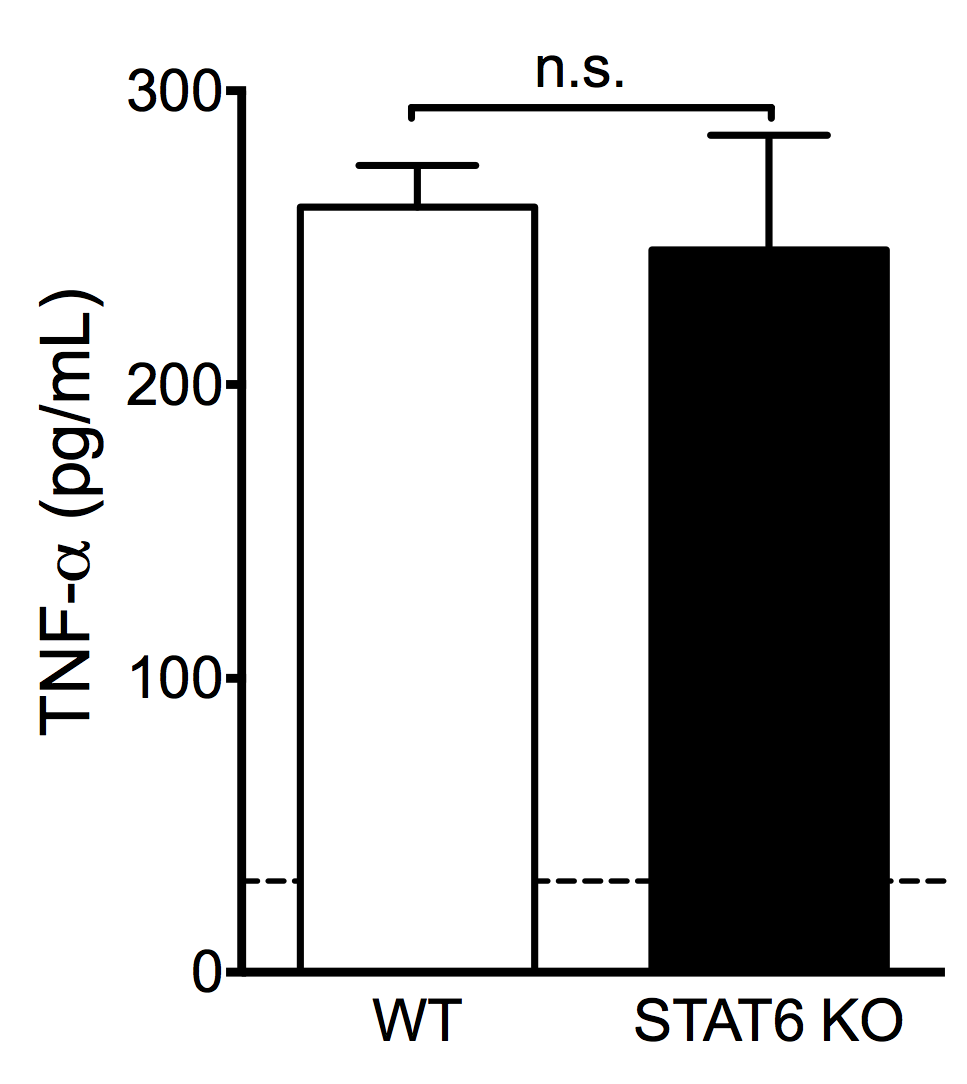

Supplement: S5 Fig — TNF-α protein amount in the lung was assessed at day 3 p.i. in WT and STAT6 KO FI-RSV-immunized mice via ELISA. Data are represented as mean ± SEM of two independent experiments (n = 8 mice total). Groups were compared using Student’s t test. (TIFF) [file ppat.1004757.s005.tiff]
